# Motion Syros: tradipitant effective in the treatment of motion sickness; a multicenter, randomized, double-blind, placebo-controlled study

**DOI:** 10.3389/fneur.2025.1550670

**Published:** 2025-03-04

**Authors:** Vasilios M. Polymeropoulos, Leah Kiely, Margaret L. Bushman, E. Blake Sutherland, Abigail R. Goldberg, Annalise X. Pham, Cameron R. Miller, Raina Mourad, Tanner R. Davis, Nikolas V. Pham, Dane B. Morgan, Abigail K. Giles, Changfu Xiao, Christos M. Polymeropoulos, Gunther Birznieks, Mihael H. Polymeropoulos

**Affiliations:** Vanda Pharmaceuticals Inc., Washington, DC, United States

**Keywords:** motion sickness, tradipitant, neurokinin-1, seasickness, seasickness prevention

## Abstract

**Introduction:**

Motion sickness has afflicted travelers since ancient times. Neurokinin-1 (NK1) receptor antagonists have therapeutic potential as treatments for the symptoms of motion sickness due to the widespread expression of NK1 receptors throughout important locations in the emetic pathway in the network of brainstem nuclei and the gut. This study evaluated the efficacy of tradipitant, a novel NK1 receptor antagonist, in preventing motion sickness symptoms in variable sea conditions.

**Methods:**

A total of 365 adult participants with a history of motion sickness embarked on boat trips under variable sea conditions. Study participants were distributed across 34 boat trips that took place between November 2021 and April 2023 in coastal waters of the United States. Participants were randomized 1:1:1 and received 170 mg tradipitant (*n* = 120), 85 mg tradipitant (*n* = 123) or placebo (*n* = 122). The symptoms of vomiting and nausea were evaluated with questionnaires every 30 min during the approximately four-hour trips. The primary efficacy endpoint for the study was the percentage of vomiting during vehicle travel. Statistical hypothesis testing was performed at the two-sided alpha level of 0.05 unless specified otherwise. Tests were declared statistically significant if the calculated *p*-value was ≤ 0.05.

**Results:**

The incidence of vomiting in both dosing arms of tradipitant was significantly lower than the placebo group across all boat trips (170 mg tradipitant = 18.3%, 85 mg tradipitant = 19.5%, placebo = 44.3%, *p* < 0.0001 for both dose comparisons against placebo). Tradipitant prevented severe nausea and vomiting as compared to participants taking placebo (tradipitant = 18.03%, placebo = 37.70%, *p* < 0.0001).

**Discussion:**

Tradipitant 170 mg and 85 mg have been confirmed to be effective in the prevention of vomiting associated with motion sickness across varied sea conditions.

**Clinical trial registration:**

ClinicalTrials.gov, identifier NCT04327661.

## Introduction

Motion sickness has afflicted travelers for thousands of years, being described in texts by the philosophers and physicians of ancient Greece (1). The ancient Greek physician Hippocrates wrote “sailing on the sea proves that motion disorders the body” (2). Habituation to motion sickness was understood in ancient times, noting that experienced sailors were much less likely to become ill at sea as compared to the occasional traveler (1). For intermittent travelers, therapeutics for motion sickness can be particularly beneficial. While there is a wide variation in motion sickness susceptibility, it has been demonstrated that given proper provocative stimuli, almost anyone can experience motion sickness under specific conditions (3).

Motion sickness is characterized by an array of symptoms with the most common and disruptive being nausea and vomiting (4, 5). Stimuli capable of triggering motion sickness are present in diverse environments spanning land, sea, air, and space (2). Regarding the mechanism of inducing motion sickness, the sensory conflict theory postulates that a discordance between actual and perceived motion leads to a physiological response and pursuant symptoms. This discordance can result from either a conflict between the visual and vestibular systems, or from a mismatch within intravestibular sensory inputs in the semicircular canals and otoliths (6).

Since antiquity, travelers have attempted to attenuate the nausea and vomiting from motion sickness by employing remedies such as mint and crushed rose petals (1). The currently approved motion sickness medications in the United States have varying degrees of efficacy and may have unpleasant, and at times dangerous, adverse effects including drowsiness, dizziness, blurred vision, and impaired operational performance (7, 8). Additionally, prolonged use of these medications may elevate the risk of developing dementia (9, 10). While the American Academy of Family Physicians (AAFP) recommends scopolamine as the leading therapy for the treatment of motion sickness, it acknowledges its limitation in the prevention of vomiting (11).

Neurokinin-1 (NK1) receptor antagonists have the potential to be efficacious as therapeutics for motion sickness. NK1 receptors are expressed centrally in the brainstem and nucleus tractus solitarius (NTS), peripherally projecting in the neurons to the antrum of the stomach, and enteric nervous system innervating the smooth muscle of the small intestine (12–16). Substance P, a member of the tachykinin family, is a neurotransmitter that binds the NK1 receptor (12, 13). Centrally, the NTS receives emetogenic signals in response to stimuli including dizziness and vertigo to trigger vomiting (14). A combination of the substance P mediated systems may contribute to the nausea and vomiting in motion sickness.

Tradipitant is an NK1 receptor antagonist that has demonstrated potential as an effective therapeutic for motion sickness. In a previous study, Motion Sifnos, 126 participants attended boat trips lasting approximately 4 h on the Pacific Ocean, where they were randomized to receive placebo or tradipitant 170 mg. Participants who received tradipitant had a significantly lower incidence of vomiting as compared to those on placebo across all boat trips (tradipitant = 17.5%, placebo = 39.7%, *p* = 0.0039). Tradipitant has also shown potential in the treatment of nausea and vomiting in patients with gastroparesis (17, 18).

This study, Motion Syros, was designed to further investigate the efficacy of tradipitant for the treatment of motion sickness. Motion Syros evaluated tradipitant in over 350 participants under varied sea conditions in the Atlantic Ocean and Gulf of Mexico in addition to the Pacific Ocean. To understand whether a lower dose of tradipitant may be efficacious, a dosing arm of 85 mg was included in addition to 170 mg.

## Methods

The Motion Syros study (NCT04327661) was a randomized, double-blind, placebo-controlled study to investigate the efficacy and safety of a single 85 mg or 170 mg oral dose of tradipitant in participants affected by motion sickness during vehicle travel.

### Participants

Eligible participants were adult men and women aged between 18 and 75 years with a history of motion sickness, otherwise in good health (as determined by medical and psychiatric history, physical examination, electrocardiogram, serum chemistry, and hematology), and without a condition causing chronic nausea. All participants provided written informed consent. Ethical oversight of the study procedures was conducted by the Institutional Review Board at Advarra Inc. Many participants had described sea travel to exacerbate their symptoms of motion sickness most severely. Recruitment of participants was accomplished through advertisements, participant databases, and pre-screening interviews conducted either online or by phone script.

**Figure 1:**
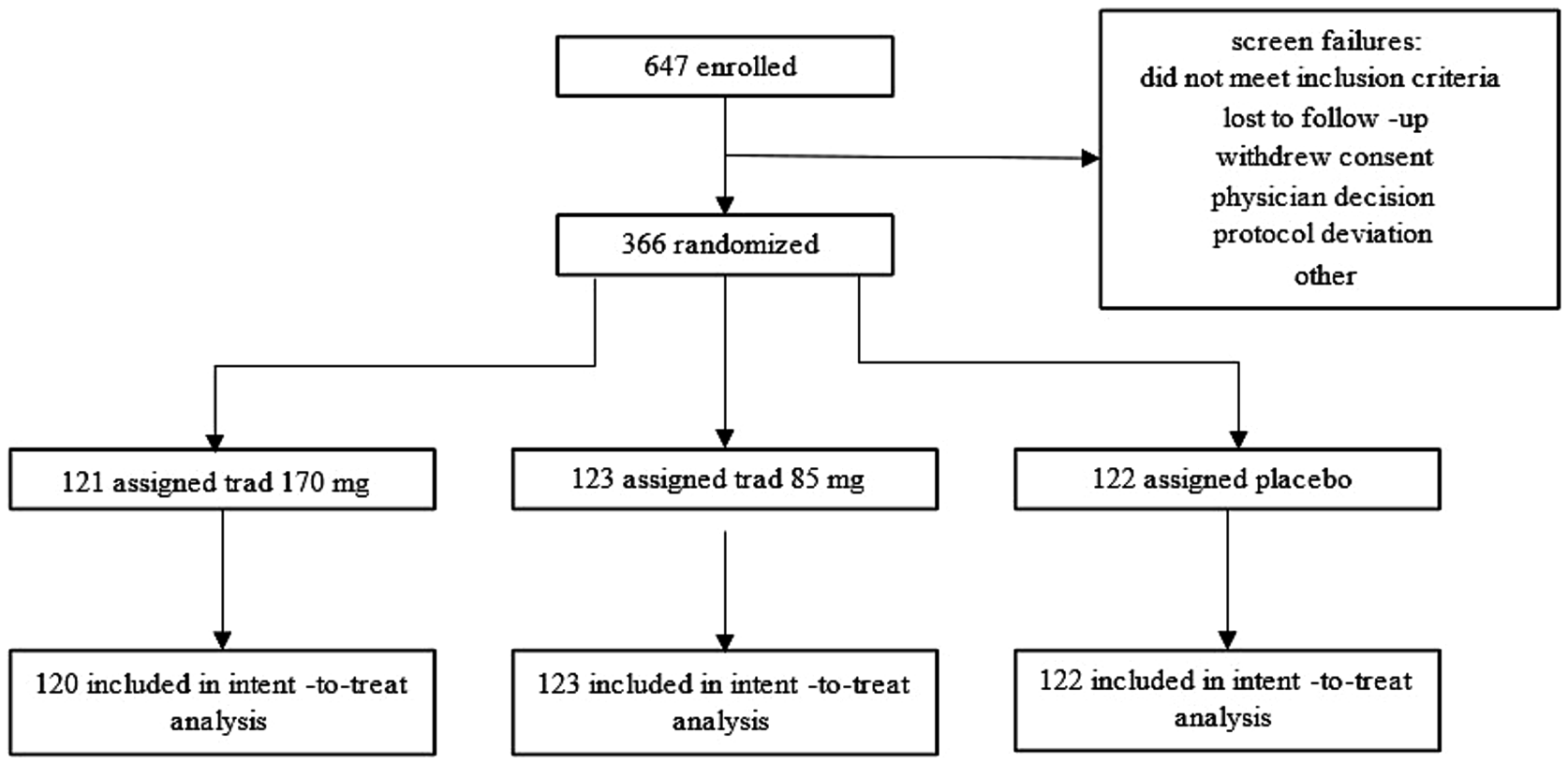
Participant disposition flow chart. The flow chart illustrates how participants entered and completed the Motion Syros study.

Interested participants opted in by completing a Motion Sickness Eligibility Questionnaire (MSEQ) either online or over the phone. Based on the severity of motion sickness symptoms, potential participants were contacted for a phone interview to understand their history of motion sickness. To demonstrate the protective effects of tradipitant, participants were deemed eligible if they had a signification history of motion sickness symptoms including vomiting and thus would have the most potential to demonstrate an improvement with treatment. A total of 647 participants were enrolled in the study and screened for eligibility. 366 of those participants met screening criteria and were randomized to take part in the vehicle travel assessment (Figure 1). Participants, in groups of one to 35, took part in one of 34 boat trip travel assessments between November 2021 and April 2023. These boat trips occurred under variable sea conditions in the Atlantic Ocean (near Boston, New York City, and Miami), the Gulf of Mexico (near Fort Myers and Tampa), and in the Pacific Ocean (near Los Angeles and San Diego). Boat trip groups were organized based on time and availability of participants, clinical site staff, and nautical professionals, as well as local weather conditions.

### Procedures

At the screening visit (V1), safety assessments, including medical history, physical exam, electrocardiogram, and laboratory tests, were performed to assess participants eligibility after the informed consent form was signed. Critical screening criteria included a reported history of motion sickness and no history of chronic nausea caused by a disorder such as irritable bowel syndrome, gastroparesis, or cyclic vomiting syndrome. Participants who did not meet eligibility criteria were considered screen failures. All participants completed the vehicle travel assessment in a boat. Prior to Visit 2 (V2), a wave height assessment was performed based on NOAA sea conditions to determine if the travel assessment would take place. If the wave height was predicted to be above 1 meter, then the travel assessment would proceed as planned. If the wave height was predicted to be below 1 meter, the travel assessment would be rescheduled. No vehicle travel assessments were rescheduled.

Upon arrival on the day of the travel assessment (V2), participants were randomly assigned to 1 of 3 treatment groups (1:1:1 ratio) stratified by site through an Interactive Web Response System. Participants were administered a single oral dose identical in appearance of either 85 mg tradipitant, 170 mg tradipitant, or placebo approximately 60 min prior to entering the boat for the travel assessment. Collection of adverse event information began after administration of tradipitant. Each participant attended a single boat trip lasting between 125 and 265 min under variable sea conditions with peak wave heights ranging from 0.5 to 2.5 meters. Average wind speed, peak wave period, and peak wave height were also recorded for each trip. All boats were privately chartered with a full staff of nautical professionals and had indoor seating cabins with outside visibility. All boats were United States Coast Guard certified and varied from deep sea fishing charters to double-decker ferries, ranging from approximately 65–110 feet in length. The study was designed to expose participants to sea conditions expected to elicit symptoms of motion sickness and thus display the protective effects of tradipitant in a variety of real-world conditions.

On the boat, participants were instructed to remain in assigned seats inside the boat and refrain from exiting the main cabin of the boat to any exterior standing decks. Every attempt was made to provide adequate space between assigned seats to limit the potential effect of a participant’s motion sickness symptoms on others. Participants were prohibited from sleeping for the duration of the boat trip to complete the assessments. At their seats, participants had access to snacks including water, juice, and crackers, as well as disposable emesis bags, wipes, and tissues in case of vomiting. There was access to restrooms on the vessels.

**Figure 2:**
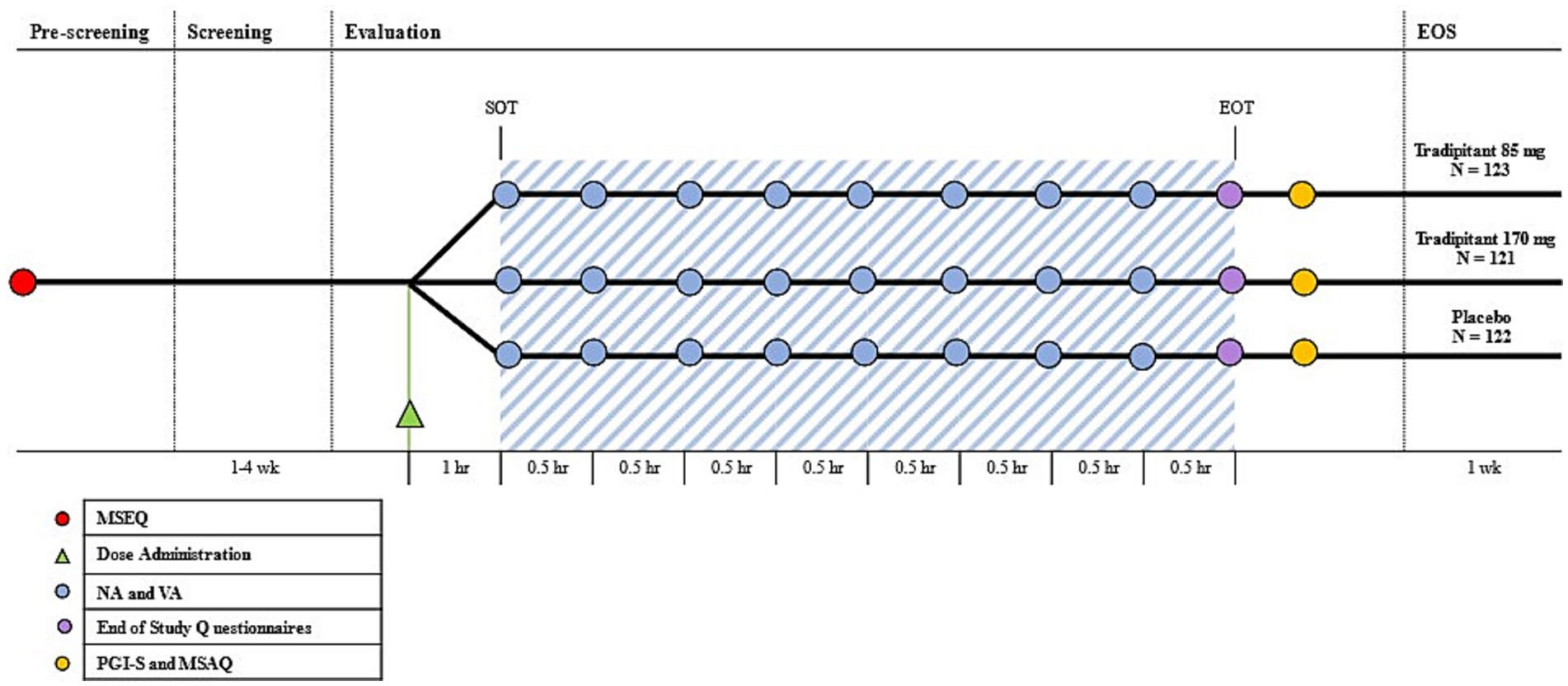
Motion Syros study design. The Motion Sickness Eligibility Questionnaire (MSEQ, red circle) was used to pre-screen prospective participants for self-reported histories of motion sickness. Approximately 60 min prior to the start of travel (SOT), participants were randomized to either tradipitant 85 mg, tradipitant 170 mg, or placebo (Dose Administration, green triangle). The blue shaded area represents boat travel. The Nausea Assessment and Vomiting Assessment were administered every 30 min (NA and VA, blue circle). The end of study questionnaires consisting of an additional NA and VA were administered upon the end of travel (EOT, purple circle). After the completion of boat travel (EOT), participants were instructed to complete the Patient Global Impression of Severity and the Motion Sickness Assessment Questionnaire (PGI-S and MSAQ, yellow circle). The end of study visit occurred 1 week after the boat trip (EOS).

Every 30 min throughout the duration of the boat trip, participants were instructed to complete both the Vomiting Assessment (VA) and Nausea Assessment (NA) to assess their symptoms of motion sickness (Figure 2). The VA is a 1-item questionnaire, where participants indicate whether or not they have vomited, in order to objectively measure the incidence of emesis. The NA is a 5-point scale rated 0–4 where participants select the number that most represents the severity of their nausea symptoms ranging from none to very severe.

At the completion of vehicle travel, participants completed additional questionnaires including the Motion Sickness Assessment Questionnaire (MSAQ) and the Patient Global Impression of Severity Questionnaire (PGI-S) for motion sickness (Figure 2). The MSAQ is a 16-statement questionnaire to assess motion sickness, which can be subdivided into affected body systems. Participants rate each statement 1–9, ranging from low severity to high severity (19). The PGI-S for motion sickness is a 5-item scale rated 0–4 where participants indicate the severity of their motion sickness symptoms ranging from none to very severe. Within 7 days of the vehicle travel assessment, participants returned to the clinical site for end-of-study safety assessments (Visit 3-V3).

### Statistical analysis

The primary efficacy endpoint for the study was percentage of vomiting during vehicle travel as assessed by the VA questionnaire. The percentage of vomiting was assessed by a Cochran–Mantel–Haenszel (CMH) test with adjusting for pooling port. The pooling port corresponded to the location of the site. The effects of tradipitant on nausea severity as measured by the NA and on the symptoms of motion sickness as measured by the MSAQ and PGI-S were analyzed using an analysis of covariance (ANCOVA) model with the main effects of treatment group, pooling port, and wave height. A sample size of 100 participants per arm (300 total) provided around 96% power to detect a 26% difference in vomiting between tradipitant and placebo assuming 50% of participants on placebo will vomit and 24% of participants on tradipitant will vomit.

Given the established relationship between the magnitude of vertical oscillation of the stimulus and motion sickness severity, subgroup analyses were conducted of participants exposed to sea conditions classified as either calm (wave height < 1 m) or rough (wave height ≥ 1 m) (20, 21). Primary efficacy analysis for vomiting incidence was conducted in these subgroups.

Statistical hypothesis testing was performed at the two-sided alpha level of 0.05 unless specified otherwise. Tests were declared statistically significant if the calculated *p*-value was ≤ 0.05.

## Results

Of the 366 participants randomized, 365 participants administered study medication and 364 completed the study. The two participants who withdrew from the study were randomized to the 170 mg tradipitant group and both withdrew consent to participate in the study. Baseline demographic characteristics for the study population are reported in Table 1. Demographics and baseline characteristics were similar across all three treatment groups. Participants were of a diverse background in terms of age, ethnicity, and physical characteristics, representative of the general US population. Females made up the majority of the study population (66.7%), which was to be expected based on epidemiological knowledge about the differences in motion sickness susceptibility between males and females (21–23).

**Table 1:** Demographics and baseline characteristics for study population.

	Placebo (*N* = 122)	Tradipitant 170 mg (*N* = 121)	Tradipitant 85 mg (*N* = 123)	Total (*N* = 366)
Sex, *n* (%)				
Male	47 (38.5)	34 (28.1)	41 (33.3)	122 (33.3)
Female	75 (61.5)	87 (71.9)	82 (66.7)	244 (66.7)
Race, *n* (%)				
American Indian or Alaskan native	2 (1.6)	1 (0.8)	0 (0.0)	3 (0.8)
Asian	11 (9.0)	7 (5.8)	9 (7.3)	27 (7.4)
Black or African American	5 (4.1)	10 (8.3)	5 (4.1)	20 (5.5)
Native Hawaiian or Pacific Islander	0 (0.0)	0 (0.0)	1 (0.8)	1 (0.3)
White	102 (83.6)	103 (85.1)	105 (85.4)	310 (84.7)
Other	2 (1.6)	0 (0.0)	3 (2.4)	5 (1.4)
Ethnicity, *n* (%)				
Hispanic or Latino	14 (11.5)	14 (11.6)	12 (9.8)	40 (10.9)
Not Hispanic or Latino	105 (86.1)	99 (81.8)	102 (82.9)	306 (83.6)
Not Reported	3 (2.5)	8 (6.6)	8 (6.5)	19 (5.2)
Other	0 (0.0)	0 (0.0)	1 (0.8)	1 (0.3)
Age, years				
Mean (SD)	48.6 (13.48)	47.7 (14.84)	49.6 (14.40)	48.6 (14.23)
Min, Max	19, 75	21, 75	21, 75	19, 75
Baseline BMI, kg/m^2^				
Mean (SD)	28.04 (5.184)	26.79 (4.633)	27.30 (4.927)	27.38 (4.934)
Min, Max	18.92, 44.42	19.06, 38.79	18.68, 39.63	18.68, 44.42

Baseline is defined as the last non-missing value recorded prior to the first dose of the study drug. Data are *n* (%) or mean (SD).

Each participant attended a single boat trip lasting between 125 and 265 min each. Participants attended 34 separate vehicle travel assessments in groups of one to 35. Summary statistics for sea conditions by boat trip and number of participants are reported by site in Table 2. The range for peak wave height across trips was between 0.50 and 2.50 meters and the range for average wind speed was between 2.0 and 26.1 knots.

**Table 2:** Summary of sea conditions by boat trip.

Trip date	Location	Participants (*n*)	Duration (min)	Peak wave height (m)	Peak wave period (sec)	Average wind speed (knots)	Wind direction (N, S, E, W)
November 15th, 2021	San Diego	5	237	1.50	10	6.0	WNW
February 5th, 2022	Tampa	3	171	1.20	4	17.0	NW
March 6th, 2022	Miami	7	125	1.40	6	17.0	NNE
March 13th, 2022	Tampa	4	168	1.20	3	2.0	N
March 14th, 2022	San Diego	6	168	1.19	14	15.0	W
April 2nd, 2022	Los Angeles	14	248	1.20	10	6.0	SW
April 21st, 2022	San Diego	3	216	1.20	10	10.0	WNW
April 23rd, 2022	Fort Myers	5	223	0.64	4	22.1	NE
April 23rd, 2022	Los Angeles	5	175	1.71	13	13.0	W
May 22nd, 2022	Tampa	1	240	1.00	2	15.0	SE
June 3rd, 2022	San Diego	7	213	1.07	9	6.0	WSW
July 8th, 2022	Los Angeles	14	240	0.61	8	9.0	WSW
July 11th, 2022	San Diego	8	228	1.07	6	10.0	WNW
July 16th, 2022	New York	3	180	1.30	10	9.0	SE
July 31st, 2022	Miami	3	155	1.60	3	16.0	SE
September 10th, 2022	Los Angeles	16	230	1.58	8	16.0	SSE
September 17th, 2022	San Diego	11	208	1.20	5	11.0	SW
October 15th, 2022	New York	4	160	2.10	8	8.0	S
November 4th, 2022	San Diego	8	193	1.20	7	10.0	NW
December 3rd, 2022	Los Angeles	18	233	0.82	11	9.0	W
December 3rd, 2022	Tampa	9	216	0.50	2	7.0	NNE
December 10th, 2022	New York	17	195	1.83	12	17.0	N/NE
January 21st, 2023	New York	15	265	0.61	8	6.0	NNW
January 28th, 2023	Boston	14	180	1.07	5	26.1	WSW
January 28th, 2023	Tampa	3	193	1.20	4	14.0	NE
February 4th, 2023	Los Angeles	12	239	1.22	8	5.0	N/NE
February 4th, 2023	San Diego	17	208	1.06	14	10.0	NW
March 11th, 2023	Los Angeles	18	194	1.50	7	5.0	NW
March 18th, 2023	New York	35	180	1.83	12	20.0	NW
March 25th, 2023	Tampa	5	220	1.20	3	15.0	SSW
April 1st, 2023	New York	24	150	2.50	5	25.0	WSW
April 1st, 2023	Boston	21	180	1.10	7	6.0	SW
April 1st, 2023	Los Angeles	16	205	1.30	5	12.0	W
April 1st, 2023	San Diego	14	192	1.70	8	9.0	WNW

Wind direction defined as N, North; S, South; E, East; W, West.

**Figure 3:**
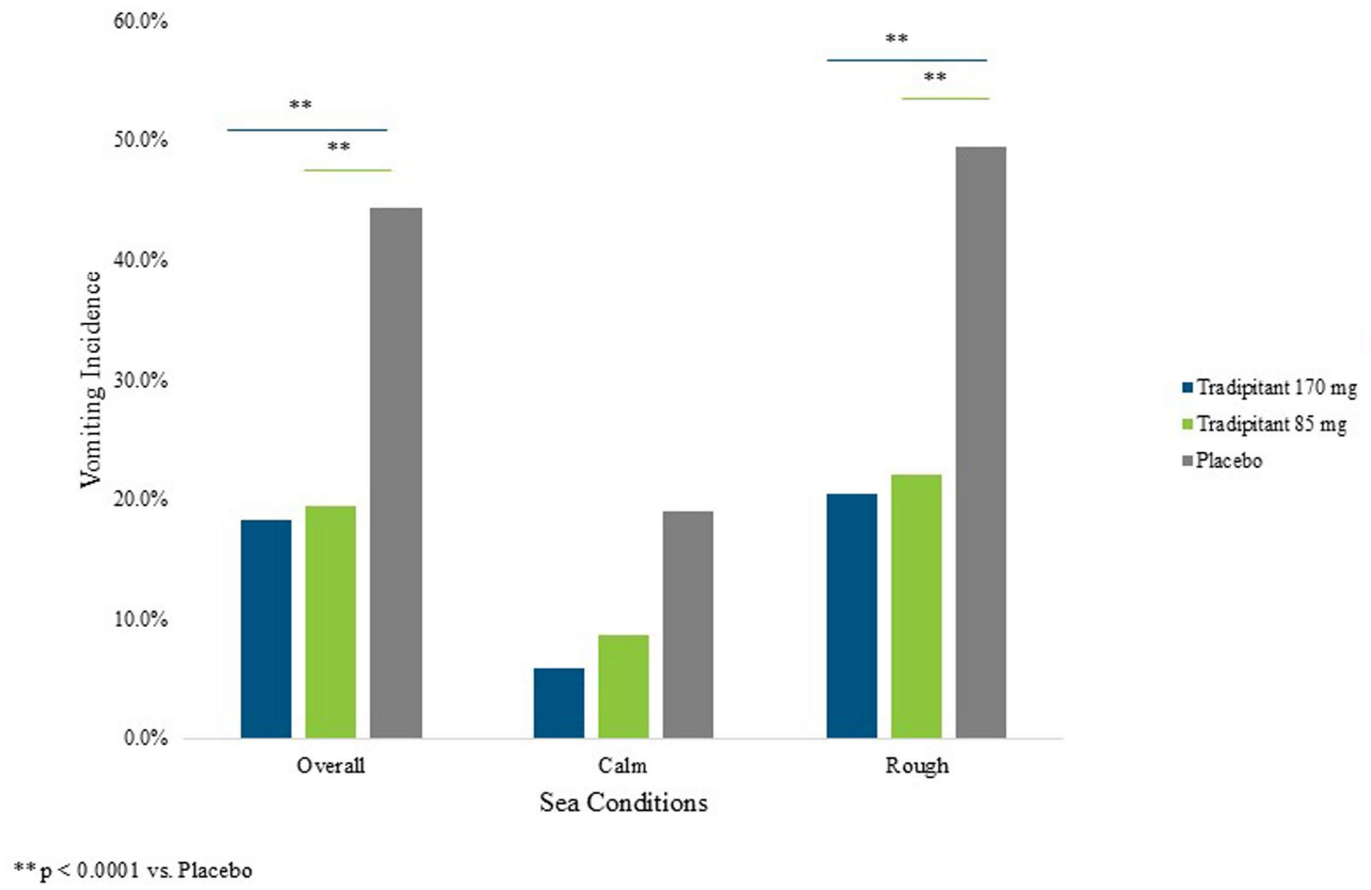
Percentage of vomiting by sea conditions. The figure reflects the percentage of participants vomiting (vomiting incidence) across different sea conditions. A participant was considered as having vomited if they ever marked “Yes” on the VA questionnaire.

A summary of assessments for the intent-to-treat (ITT) population is reported in Table 3. The incidence of vomiting in both tradipitant groups was significantly lower than the placebo group across all boat trips (Figure 3). In the placebo group, 44.3% of participants (54/122) vomited as compared to 18.3% of participants (22/120) in the 170 mg tradipitant group (*p* < 0.0001) and 19.5% of participants (24/123) in the 85 mg tradipitant group (*p* < 0.0001). Treatment with 170 mg and 85 mg tradipitant resulted in reduction of risk (RRR) of vomiting of over 50% (170 mg RRR = 58.7%, 85 mg RRR = 56.0%).

**Table 3:** Summary of primary endpoints for the ITT Population.

Primary endpoints	Placebo (*N* = 122)	Tradipitant 170 mg (*N* = 120)	Tradipitant 85 mg (*N* = 123)
Overall vomiting, n/N (%)	54/122 (44.3)	22/120 (18.3)	24/123 (19.5)
*p*-value (vs Placebo)^a^		< 0.0001	< 0.0001
Risk difference for vomiting (95% CI)^b^		−0.26 (−0.371, −0.147)	−0.25 (−0.360, −0.135)
Adjusted relative risk for vomiting (95% CI)^c^		0.42 (0.272, 0.638)	0.44 (0.293, 0.655)
More than one instance of vomiting, n/N (%)	38/122 (31.1)	13/120 (10.8)	17/123 (13.8)
*p*-value (vs Placebo)^a^		0.0001	0.0011
Risk difference for vomiting (95% CI)^b^		−0.20 (−0.302, −0.104)	−0.17 (−0.276, −0.071)
Adjusted relative risk for vomiting (95% CI)^d^		0.35 (0.196, 0.623)	0.44 (0.264, 0.736)
Vomiting, n/N (%) in Rough Seas (Wave height ≥ 1 m)	50/101 (49.5)	21/103 (20.4)	22/100 (22.0)
*p*-value (vs Placebo)^a^		< 0.0001	< 0.0001
Risk difference for vomiting (95% CI)^b^		−0.29 (−0.416, −0.166)	−0.28 (−0.402, −0.148)
Adjusted relative risk for vomiting (95% CI)^e^		0.41 (0.269, 0.638)	0.43 (0.289, 0.655)

a Cochran–Mantel–Haenszel test adjusts for the pooling port.

b Wald Interval.

c Adjusted relative risk and 95% CI are based on the ratio of vomiting rates for tradipitant vs. placebo, stratified by pooling port.

d Adjusted relative risk and 95% CI are based on the ratio of percentages of participants with more than one instance of vomiting for tradipitant vs. placebo, stratified by pooling port.

e Adjusted relative risk and 95% CI are based on the ratio of overall vomiting rates for tradipitant vs. placebo, stratified by pooling port.

**Figure 4:**
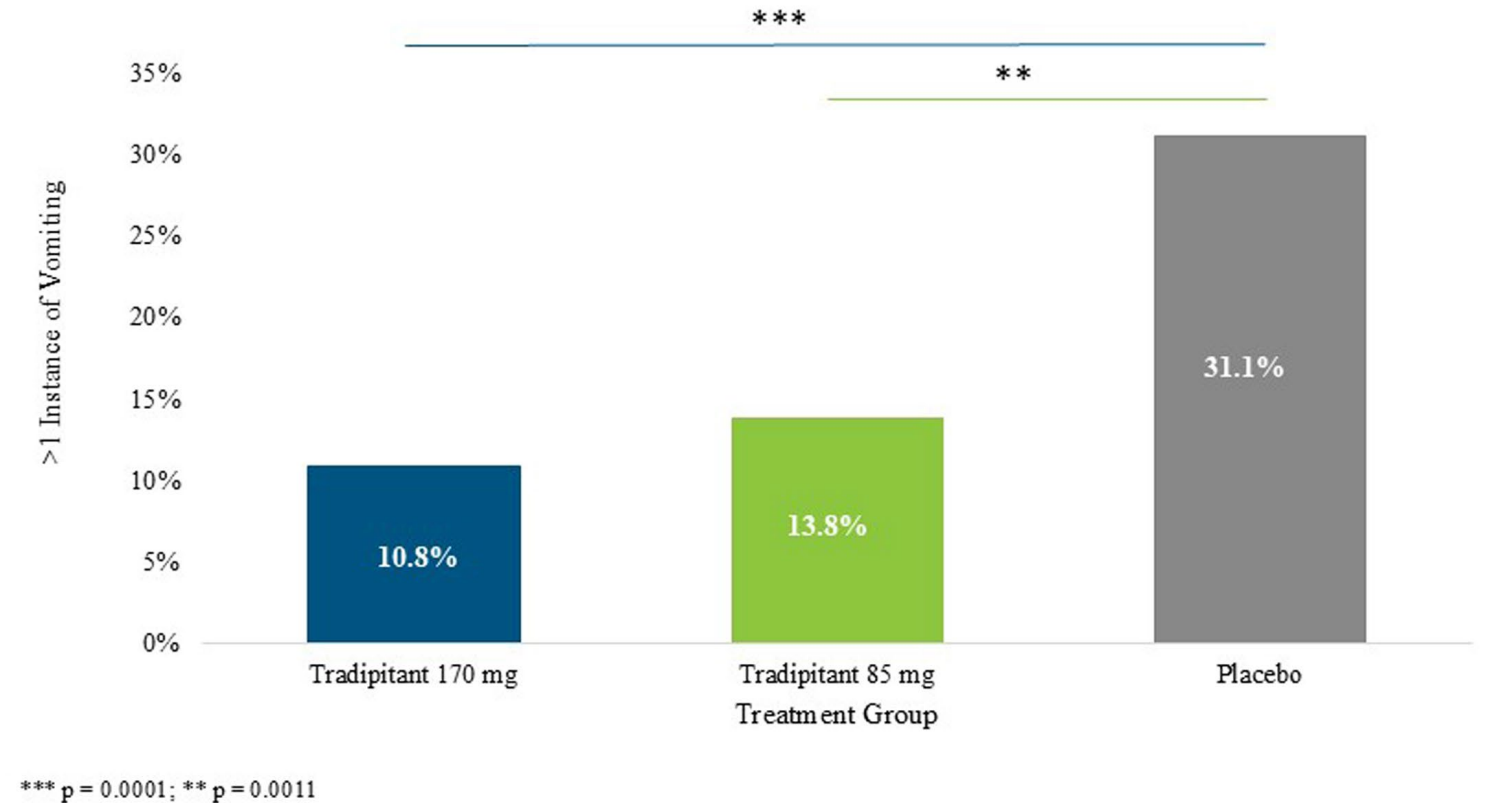
Percentage of more than one incidence of vomiting. The figure depicts the percentage of participants who vomiting more than once (> 1 instance of vomiting) across different sea conditions. A participant was considered to have vomited more than once if they marked “Yes” on the VA questionnaire at more than one 30-min interval.

Multiple instances of vomiting were classified as participants that answered “yes” to the VA on multiple questionnaires. Both 170 mg and 85 mg tradipitant doses were shown to be superior to placebo in preventing more than one incidence of vomiting (*p* = 0.0001, *p* = 0.0011, respectively) (Figure 4). Participants on 170 mg tradipitant had a 20.0% lower incidence of repeated vomiting episodes as compared to placebo (10.8% vs. 31.1%, 95% CI: −0.302, −0.104), while those on 85 mg tradipitant had a 17% lower incidence of repeated vomiting as compared to participants on placebo (13.8% vs. 31.1%, 95% CI: −0.276, −0.071).

Given that motion sickness severity is related to the intensity of the stimulus, and that intensity of the stimulus was variable across boat trips, sea conditions were examined in the analysis of efficacy. When considering trips with wave heights ≥1 m, the incidence of vomiting in the 170 mg tradipitant group (20.4%, *p* < 0.0001) and the 85 mg tradipitant group (22%, *p* < 0.0001) was significantly lower as compared to the placebo group (49.5%, 170 mg risk difference = −0.29, 95% CI: −0.416, −0.166, 85 mg risk difference = −0.28, 95% CI: −0.402, −0.148). In these boat trips in rough seas, participants taking tradipitant 170 mg and 85 mg reduced the risk of vomiting by 59% and 57% in each group, respectively, (Figure 3).

A summary of the secondary endpoints for this study are summarized in Table 4. In the ITT population, no statistically significant differences between treatment groups were observed for the NA, MSAQ total and subscales, and PGI-S. However, there was a slight trend toward favoring both tradipitant treatment groups over placebo. The worst nausea severity, as assessed by the NA questionnaire during travel, was lower in the 170 mg tradipitant group (LS mean difference = −0.1, 95% CI: −0.40, 0.24, *p* = 0.6110) compared to placebo, and there was no difference in the 85 mg tradipitant group (LS mean difference = 0.0, 95% CI: −0.27, 0.36, *p* = 0.7913) compared to placebo. The MSAQ gastrointestinal subscale score was lower in the 170 mg tradipitant group (LS mean difference = −3.7, 95% CI: −12.29, 4.98, *p* = 0.4058) and in the 85 mg tradipitant group (LS mean difference = −1.4, 95% CI: −9.92, 7.19, *p* = 0.7540) compared to placebo, although these differences did not reach significance. PGI-S scores were also lower in the 170 mg tradipitant group (LS mean difference = −0.1, 95% CI: −0.42, 0.23, *p* = 0.5542) and the 85 mg tradipitant group (LS mean difference = −0.2, 95% CI: −0.49, 0.15 *p* = 0.3025) compared to placebo, but did not reach statistical significance.

**Table 4:** Summary of secondary endpoints for the ITT population.

Secondary endpoints	Placebo (*N* = 122)	Tradipitant 170 mg (*N* = 120)	Tradipitant 85 mg (*N* = 123)
Worst nausea severity (0–4)	*n* = 122	*n* = 120	*n* = 123
LS mean (SE)^a^	2.5 (0.13)	2.4 (0.13)	2.6 (0.13)
Differences in LS means (95% CI)^b^		−0.1 (−0.40, 0.24)	0.0 (−0.27, 0.36)
*p*-value		0.6110	0.7913
Overall MSAQ Score (0–100)	*n* = 122	*n* = 118	*n* = 122
LS mean (SE)^a^	45.1 (2.50)	46.0 (2.53)	49.2 (2.50)
Differences in LS means (95% CI)^b^		0.9 (−5.34, 7.17)	4.1 (−2.06, 10.33)
*p*-value		0.7746	0.1899
MSAQ-GI Subscale Score (0–100)	*n* = 122	*n* = 118	*n* = 122
LS mean (SE)^a^	61.2 (3.46)	57.6 (3.49)	59.9 (3.46)
Differences in LS means (95% CI)^b^		−3.7 (−12.29, 4.98)	−1.4 (−9.92, 7.19)
*p*-value		0.4058	0.7540
PGI-S Score (0–4)	*n* = 122	*n* = 119	*n* = 123
LS mean (SE)^a^	2.2 (0.13)	2.1 (0.13)	2.0 (0.13)
Differences in LS means (95% CI)^b^		−0.1 (−0.42, 0.23)	−0.2 (−0.49, 0.15)
*p*-value		0.5542	0.3025

a LS means, CIs, and *p*-values are based on ANCOVA model with the main effects of pooling port, wave height, and treatment group.

b Difference between tradipitant and placebo.

A *post hoc* analysis was conducted to determine the effect of tradipitant on the prevention of severe nausea and vomiting (Table 5). Severe nausea and vomiting was defined as the percentage of participants with at least one incidence of vomiting and severe nausea (worst nausea ≥3 as measured on the NA). Overall, tradipitant (85 and 170 mg together) was effective in the prevention of severe nausea and vomiting (tradipitant 18.03%, placebo 37.70%, *p* < 0.0001). By treatment group, the percentage of participants who experienced severe nausea and vomiting was significantly lower in both the 170 mg tradipitant group (16.53%, *p* = 0.0003) and the 85 mg tradipitant group (19.51%, *p* = 0.0014), as compared to placebo (37.70%) (Table 5).

**Table 5:** *Post hoc* analysis of severe nausea and vomiting during vehicle travel by treatment group.

*Post hoc* analysis	Placebo (*N* = 122)	Tradipitant 170 mg (*N* = 121)	Tradipitant 85 mg (*N* = 123)	Overall tradipitant (*N* = 244)
Severe nausea^a^ and vomiting, n/N (%)	46/122 (37.70)	20/121 (16.53)	24/123 (19.51)	44/244 (18.03)
*p*-value (vs Placebo)^b^		0.0003	0.0014	< 0.0001

a Severe Nausea defined as worst nausea ≥ 3 in tradipitant treatment groups.

b Cochran–Mantel–Haenszel statistics.

The primary efficacy results were examined across participant demographic subgroups (sex, age, and BMI) for both the 85 mg and 170 mg doses (Table 6). There was no significant difference in the rate of vomiting when examined by sex for participants taking tradipitant 170 mg (females = 19.5%, males = 15.2%, *p* = 0.5979), tradipitant 85 mg (females = 23.2%, males = 12.2%, *p* = 0.1672), or placebo (females = 50.7%, males = 34.0%, *p* = 0.1437). For females, tradipitant offered significant protection against vomiting in both the 170 mg (*p* < 0.0001) and 85 mg (*p* = 0.0004) dose groups as compared to placebo. In males, the incidence of vomiting was lower in both tradipitant groups as compared to placebo but only reached statistical significance in the 85 mg (*p* = 0.0117) dose group and not 170 mg (*p* = 0.0632) compared to placebo.

**Table 6:** Demographic subgroup analysis of overall vomiting.

Demographic subgroup	Statistics	Placebo (*N* = 122)	Tradipitant 170 mg (*N* = 120)	Tradipitant 85 mg (*N* = 123)
Sex				
Male	Overall vomiting, n/N (%)	16/47 (34.0)	5/33 (15.2)	5/41 (12.2)
	*p*-value (vs Placebo)^a^		0.0632	0.0117
	Risk difference (95% CI)^b^		−0.19 (−0.371, −0.006)	−0.22 (−0.387, −0.050)
	Adjusted relative risk (95% CI)^c^		0.44 (0.182, 1.084)	0.35 (0.145, 0.837)
Female	Overall vomiting, n/N (%)	38/75 (50.7)	17/87 (19.5)	19/82 (23.2)
	p-value (vs Placebo)^a^		< 0.0001	0.0004
	Risk difference (95% CI)^b^		−0.31 (−0.452, −0.171)	−0.27 (−0.420, −0.130)
	Adjusted relative risk (95% CI)^c^		0.39 (0.240, 0.640)	0.47 (0.300, 0.731)
Age (Years)				
< 50	Overall vomiting, n/N (%)	25/56 (44.6)	14/63 (22.2)	12/59 (20.3)
	p-value (vs Placebo)^a^		0.0177	0.0027
	Risk difference (95% CI)^b^		−0.22 (−0.390, −0.058)	−0.24 (−0.409, −0.077)
	Adjusted relative risk (95% CI)^c^		0.50 (0.290, 0.866)	0.43 (0.239, 0.769)
≥ 50	Overall Vomiting, n/N (%)	29/66 (43.9)	8/57 (14.0)	12/64 (18.8)
	*p*-value (vs Placebo)^a^		0.0005	0.0015
	Risk difference (95% CI)^b^		−0.30 (−0.449, −0.149)	−0.25 (−0.405, −0.099)
	Adjusted relative risk (95% CI)^c^		0.32 (0.158, 0.658)	0.41 (0.232, 0.736)
BMI (kg/m^2^)				
< 30	Overall vomiting, n/N (%)	33/78 (42.3)	16/93 (17.2)	15/90 (16.7)
	*p*-value (vs Placebo)^a^		0.0003	0.0002
	Risk difference (95% CI)^b^		−0.25 (−0.385, −0.117)	−0.26 (−0.390, −0.122)
	Adjusted relative risk (95% CI)^c^		0.41 (0.242, 0.679)	0.39 (0.229, 0.657)
≥ 30	Overall vomiting, n/N (%)	21/44 (47.7)	6/27 (22.2)	9/33 (27.3)
	*p*-value (vs Placebo)^a^		0.0830	0.0830
	Risk difference (95% CI)^b^		−0.26 (−0.470, −0.040)	−0.20 (−0.416, 0.007)
	Adjusted relative risk (95% CI)^c^		0.53 (0.238, 1.163)	0.58 (0.311, 1.079)

a *p-value is based on CMH test with adjusting for the pooling port.*

b Wald Interval.

c Adjusted relative risk and 95% CI are based on the ratio of overall vomiting rates for tradipitant vs. placebo, stratified by the pooling port.

There was no significant difference in the rate of vomiting when examined by age for participants taking tradipitant 170 mg (age < 50 = 22.2%, age ≥ 50 = 14.0%, *p* = 0.4248), tradipitant 85 mg (age < 50 = 20.3%, age ≥ 50 = 18.8%, *p* = 0.71), or placebo (age < 50 = 44.6%, age ≥ 50 = 43.9%, *p* = 0.7833). Participants <50 years of age and ≥ 50 years of age had significantly lower incidences of vomiting on both doses of tradipitant.

When examined by BMI, there was no significant difference in the rate of vomiting for participants taking tradipitant 170 mg (BMI < 30 = 17.2%, BMI ≥ 30 = 22.2%, *p* = 0.5963), tradipitant 85 mg (BMI < 30 = 16.7%, BMI ≥ 30 = 27.3%, *p* = 0.2451), or placebo (BMI < 30 = 42.3%, BMI ≥ 30 = 47.7%, *p* = 0.7112). For participants with a BMI < 30, tradipitant offered significant protection against vomiting in the 170 mg (*p* = 0.0003) and 85 mg (*p* = 0.0002) dose groups as compared to placebo. In participants with a BMI ≥ 30 the incidence of vomiting was lower in both tradipitant groups as compared to placebo, but did not reach statistical significance in the 170 mg (*p* = 0.0830) or 85 mg (*p* = 0.0830) dose groups as compared to placebo.

Tradipitant was well tolerated during the study, with an overall incidence of treatment emergent adverse events that was similar across the two tradipitant groups (170 mg tradipitant = 28.3%, 85 mg tradipitant = 26.8%) compared to the frequency in the placebo group (9.0%). Adverse events occurring in more than 5% of participants in any treatment group are summarized in Table 7. The most reported probably or possibly related drug-related AEs were fatigue, headache, and somnolence. Each of these adverse events occurred more frequently in the two treatment groups than in the placebo group (fatigue: 170 mg tradipitant = 8.3%, 85 mg tradipitant = 4.1%, placebo = 0.0%; headache: 170 mg tradipitant = 8.3%, 85 mg tradipitant = 5.7%, placebo = 2.5%; somnolence: 170 mg tradipitant = 8.3%, 85 mg tradipitant = 6.5%, placebo = 0.8%). The most commonly reported severity of these AEs was mild. No serious adverse events were reported during study participation.

**Table 7:** Summary of treatment-emergent adverse events occurring in ≥5% of participants.

Preferred term	Total (*N* = 365)	Placebo (*N* = 122)	Tradipitant 170 mg (*N* = 120)	Tradipitant 85 mg (*N* = 123)
Number of Subjects with any TEAE	78 (21.4)	11 (9.0)	34 (28.3)	33 (26.8)
Headache	20 (5.5)	3 (2.5)	10 (8.3)	7 (5.7)
Somnolence	19 (5.2)	1 (0.8)	10 (8.3)	8 (6.5)
Fatigue	15 (4.1)	0 (0.0)	10 (8.3)	5 (4.1)

A treatment-emergent adverse event (TEAE) is defined as an adverse event that is newly occurring or worsening from treatment dosing until 3 days after the final dose of study medication. Adverse events were coded using the Medical Dictionary for Regulatory Activities (MedDRA version 23.0) coding dictionary.

## Discussion

The Motion Syros study demonstrated the robust efficacy of both doses of tradipitant in the prevention of vomiting associated with motion sickness. These results compound data from a previous study that showed tradipitant 170 mg to be superior to placebo in preventing vomiting in similar conditions. Additionally, both dosing arms of tradipitant were superior to placebo in preventing multiple episodes of vomiting (170 mg tradipitant = 10.8%, 85 mg tradipitant = 13.8%, placebo = 31.1%). This could be of significant utility given the dangers of repeated vomiting leading to dehydration and electrolyte loss (24).

In this study, we attempted to examine nausea and vomiting as separate entities through different questionnaires. In the previous study of motion sickness, we utilized the Motion Sickness Severity Scale (MSSS) which is a validated scale for the assessment of motion sickness that assesses nausea and vomiting along a continuum (25). Both dosing arms were non-superior to placebo when examining the nausea scale alone, consistent with a prior study of tradipitant in the treatment of motion sickness (4). Given that nausea and vomiting are related and thought to occur along a continuum, a limitation of this study could have been in their separate evaluation whereas we may have been better served to utilize a combined scale (26, 27). Another plausible reason that it was challenging to capture a significant difference in therapeutic effect in mild or moderate nausea may be due to the population studied. We selected individuals with a significant history of motion sickness, including past episodes of vomiting. Vomiting has been postulated to temporarily abate nausea which may confound recording nausea as a separate entity (28).

To address the potential limitation of evaluating nausea alone, a *post hoc* analysis was performed to assess the effect of tradipitant in the prevention of severe nausea and vomiting. In both dose arms tradipitant significantly prevented severe nausea and vomiting compared to placebo (170 mg tradipitant = 16.53%, *p* = 0.0003; 85 mg tradipitant = 19.51%, *p* = 0.0014; placebo = 37.70%). By combining nausea and vomiting, the core symptoms of motion sickness, we were able to capture a comprehensive view of how tradipitant helps protect against motion sickness symptoms.

The MSAQ was used to assess the full constellation of possible symptoms (nausea, vomiting, drowsiness, dizziness, queasiness, etc.) that a person may experience from motion sickness following travel. There was no significant difference between total MSAQ scores in either the 170 mg or 85 mg tradipitant groups as compared to placebo. While nausea and vomiting are the hallmark symptoms of motion sickness, the array of other symptoms that may be experienced vary greatly between individuals. Using a questionnaire that compares unique experiences between individuals may be a limitation of this study. In the future it could be desirable to establish a baseline of MSAQ for an individual and if we could expose them to the same stimulus, we could evaluate an individual against their own baseline to measure improvement. A limitation here would be the concept of habituation where an individual will acclimate to a motion sickness stimulus upon repeated exposure.

Individuals possess different levels of susceptibility to developing motion sickness. Motion sickness has been reported to affect up to 30% of the population, with females more susceptible than males (21–23, 29). The higher prevalence of motion sickness in females is reflected in the study population, with 66.7% of participants being female. When examining overall vomiting by sex, the tradipitant 170 mg group did not reach statistical significance in males. The disproportionate sample size of this group (*n* = 33) is a significant limitation to consider when interpreting this result. Females are more likely to experience severe motion sickness than males (21–23). In our study, this is shown in the placebo group where females had a greater incidence of vomiting than males (50.7 and 34.0% respectively). This could explain the lower rate of vomiting in males while still demonstrating the protective effect of tradipitant. The rate of vomiting was lower in males than females for both the tradipitant 170 mg group (males = 15.2%, females = 19.5%) and tradipitant 85 mg group (males = 12.2%, females = 23.32%). There was no statistical difference in incidence of vomiting between sex for either treatment group, further demonstrating the protective effect of tradipitant against vomiting in both males and females.

Additional analyses were conducted to examine the effect of age and BMI on the incidence of vomiting. There was no difference between participants <50 years and ≥ 50 years, and tradipitant reduced the incidence of vomiting compared to placebo in both groups. Children have increased susceptibility to motion sickness around the ages of 6–12 with a gradual decline throughout teen years and into adulthood (7, 22, 23). This study did not include pediatric participants and future research in this population with a history of motion sickness would help determine the efficacy of tradipitant in the prevention of vomiting in this population.

Regarding BMI, participants with a lower BMI (< 30) may have had increased protection against vomiting from tradipitant compared to participants with a higher BMI (≥ 30), but the small sample size in participants with a higher BMI (170 mg: *n* = 27, 85 mg: *n* = 33, placebo: *n* = 44) limits the ability to draw definitive conclusions. There is limited research on the correlation between BMI and motion sickness susceptibility, with current literature offering contradictory explanations (30, 31).

Our previous study of tradipitant in the treatment of motion sickness was conducted on boats in the Pacific Ocean and found participants were protected from vomiting during travel when administered tradipitant. This study expanded geographically as compared to the previous study, including additional bodies of water along the coasts of the United States. We elected to study tradipitant again in open seas as the continuous vertical displacement generated by waves has proven to be the most dependable stimulus of motion sickness in susceptible individuals (4, 20). This stimulus creates a discordance between actual and perceived motion, which initiates a physiological response, according to the sensory conflict theory (3, 6, 26). The concept of vertical motion defined as heave in sea travel holds true with other vehicle modalities as motion sickness is often induced during the linear accelerations of take-off and landing during air travel (32–34).

Overall, tradipitant was safe and well tolerated during the study. The incidence of treatment emergent adverse events was similar across both tradipitant treatment groups and most commonly reported event was characterized as mild in severity. This is an important distinction from currently approved therapies for motion sickness like dimenhydrinate (Dramamine), hyoscine (Scopolamine), and meclizine (Antivert) which have significant side effects. Scopolamine, the most effective available medication, has adverse effects including dry mouth, drowsiness, blurred vision, and dilation of the pupils (24, 35). Dramamine and Antivert are a part of a class of drugs known as antihistamines, which are highly sedating (8). When used as a therapy for motion sickness in naval crews, Dramamine was found to have a negative effect on psychomotor performance, by significantly impairing decision reaction time and auditory digit span (36). Meclizine can induce drowsiness and has been found to negatively impact short-term memory (37). Across both tradipitant treatment groups in our study, somnolence was reported by less than 10% of participants. Therefore, tradipitant may be beneficial for individuals with motion sickness who cannot tolerate currently approved medications’ sedative effects.

Motion sickness remains a significant burden for many travelers both in professional and leisurely settings. Further studies with tradipitant could examine its use in non-traditional modes of travel, including travel for astronauts. Given the incomplete efficacy and adverse effects of currently available therapies, tradipitant could be of utility for many travelers.

## Data Availability

The raw data supporting the conclusions of this article will be made available by the authors, without undue reservation.
